# Improved cider fermentation performance and quality with newly generated *Saccharomyces cerevisiae* × *Saccharomyces eubayanus* hybrids

**DOI:** 10.1007/s10295-017-1947-7

**Published:** 2017-04-27

**Authors:** Frederico Magalhães, Kristoffer Krogerus, Virve Vidgren, Mari Sandell, Brian Gibson

**Affiliations:** 1 VTT Technical Research Centre of Finland Ltd, Tietotie 2, P.O. Box 1000, 02044 Espoo, Finland; 2 Department of Biotechnology and Chemical Technology, School of Chemical Technology, Aalto University, Kemistintie 1, Aalto, P.O. Box 16100, 00076 Espoo, Finland; 3 Functional Foods Forum 20014 University of Turku, Itäinen Pitkäkatu 4 A, Turku, Finland

**Keywords:** *Saccharomyces eubayanus*, Cider, Hybridization, Cryotolerance

## Abstract

Yeast cryotolerance may be advantageous for cider making, where low temperatures are usually employed. Here, we crossed the cryotolerant *S. eubayanus* with a *S. cerevisiae* wine strain and assessed the suitability of the hybrids for low-temperature cider fermentation. All strains fermented the juice to 5% ABV, but at different rates; hybrid strains outperformed *S. cerevisiae*, which was sensitive to low temperatures. The best hybrid fermented similarly to *S. eubayanus*. *S. eubayanus* produced sulphurous off flavours which masked a high concentration of fruity ester notes. This phenotype was absent in the hybrid strains, resulting in distinctly fruitier ciders. Aroma was assessed by an independent consumer panel, which rated the hybrid ciders as identical to the wine strain cider. Both were significantly more pleasant than the *S. eubayanus* cider. Interspecific hybridization can apparently be used effectively to improve low-temperature fermentation performance without compromising product quality.

**Electronic supplementary material:**

The online version of this article (doi:10.1007/s10295-017-1947-7) contains supplementary material, which is available to authorized users.

## Introduction

The temperature employed in alcoholic beverages fermentations impacts the sensorial properties of the final product. There are several reasons why cold fermentations (i.e., lower than 15 °C) are preferred over warm fermentations. In brewing, these conditions allow for the production of the clean and fresh tasting lager beer, which has contributed to the global popularity of this beer style [15]. Contrarily, in white wine making, low temperature actually improves the aromatic profile of the wine [9, 10, 40]. Compounds like ethyl esters associated with fruit notes and freshness tend to be produced and maintained at higher concentrations in white wines fermented at low temperatures [40]. The reduced evaporation of volatile compounds in the cold must also be taken into consideration. Other than the sensorial properties, the use of low temperatures also reduces the likelihood of contamination by spoilage organisms, potentially obviating the use of SO_2_. It is, however, necessary to have a yeast strain that is able to survive and retain metabolic activity at these temperatures if fermentation is to proceed efficiently.

Naturally cold-tolerant strains like *Saccharomyces kudriavzevii*, *S. uvarum* or *S. eubayanus* could potentially be used for low-temperature fermentations, but tend to have higher ethanol sensitivity than *S. cerevisiae* and may, therefore, be less suitable for alcoholic fermentation [6]. It is not uncommon, however, to find hybrids of *S. cerevisiae* and a cold-tolerant *Saccharomyces* species associated with alcoholic fermentations. From the best-known case of the lager yeast *S. cerevisiae* × *S. eubayanus* hybrids [15, 26], to hybrids involving *S. cerevisiae*, *S. uvarum* and/or *S. kudriavzevii* in cider fermentations [18, 35, 42].

Since the discovery of *S. eubayanus* in Patagonia [31] there has been an interest in recreating the hybridization event and generating new lager strains for brewing applications [22, 24, 25, 37]. The discovery has also inspired new approaches to facilitate the selection of artificial hybrid strains [2]. Lager yeast hybrids have inherited the superior fermentation performance from their *S. cerevisiae* parent and the cold tolerance from *S. eubayanus*. This has resulted in strains that have superior phenotypic properties relative to their parents, a phenomenon called heterosis [15].

Cold tolerance seems the main phenotypic contribution of *S. eubayanus* to the natural and artificial lager yeast strains [22, 25, 37], but the presence of an additional high-affinity fructose transporter, Fsy1, could provide hybrids with improved ability to reduce the critical accumulation of fructose in the later phases of the wine and cider fermentations [32]. Fructose represents the larger portion of fermentable sugars in apple juice, being usually present in concentration two times higher than glucose [56].

Countries like France, Spain, Ireland and Slovenia have a strong cider culture and this is traditionally produced with naturally occurring indigenous yeast at temperatures ranging from 12 to 20 °C [41]. In the initial stages of cider production species from the genus *Candida*, *Pichia*, *Hansenula*, *Hanseniaspora* and *Metschnikowia* predominate, having a strong influence in the aromatic complexity of the cider [12, 39, 41, 55]. The alcoholic fermentation, however, is carried by *Saccharomyces* species [54].

There has been an increased interest in cider production recently, particularly in North America but also in Eastern European countries without a tradition for cider consumption. In 2015 alone, cider production, for example, grew by 106% in Poland. This accelerated production was most likely a result of a search for an alternative use for apples after the Russian ban on apple importing from Poland combined with record production levels and decrease in excise duties [17]. The increased popularity of cider can also be attributed to consumer tastes changing towards less-bitter drinks, with the addition of being gluten-free and lower in alcohol than wine.

In this work, we describe the generation of new interspecific hybrids between an *S. cerevisiae* wine strain and *S. eubayanus* type strain and characterised the potential of these hybrids for cider fermentations. The ciders were analysed for their aromatic properties and were also evaluated by a consumer panel.

## Materials and methods

### Strains

Yeast strains used in this work are listed in Table 1. Parental strains include *S. eubayanus* type strain (CBS 12357) deposited at VTT culture collection as C-12902 and *S. cerevisiae* 59A [3], a haploid isolate of the industrial wine strain Lalvin EC1118. The six hybrid strains generated by crossing the two parental strains are deposited at VTT Culture Collection as VTT C-16962 to VTT C-16967. Abbreviated codes (C962–C967) are use in the text.

**Table 1:** List of strains used in this study

Strain codes	Species	Ploidy	MtDNA	Additional information
59A	*S. cerevisiae*	1.01 *n*		Haploid derivative of industrial wine strain Lalvin EC1118 [3]
VTT C-12902 (C902)	*S. eubayanus*	2.07 *n*		*S. eubayanus* type strain; CBS12357 [31]
VTT C-16962 (C962)	Interspecies hybrid	2.29 *n*	*S. cerevisiae*	59A × C902 hybrid
VTT C-16963 (C963)	Interspecies hybrid	2.17 *n*	*S. cerevisiae*	59A × C902 hybrid
VTT C-16964 (C964)	Interspecies hybrid	2.07 *n*	*S. cerevisiae*	59A × C902 hybrid
VTT C-16965 (C965)	Interspecies hybrid	2.38 *n*	n.d.	59A × C902 hybrid
VTT C-16966 (C966)	Interspecies hybrid	2.12 *n*	n.d	59A × C902 hybrid
VTT C-16967 (C967)	Interspecies hybrid	2.25 *n*	n.d.	59A × C902 hybrid

n.d.- not detected

### Hybrid generation

Prior to hybridization, natural lysine auxotrophic mutants of *S. cerevisiae* 59A were selected based on their ability to grow on α-aminoadipic acid-containing agar [57]. Lack of growth in minimal selection medium confirmed the auxotrophy [25].

Hybrids between *S. cerevisiae* 59A *lys*- and *S. eubayanus* C902 were generated by rare mating as described in Ref. [25]. A fresh colony of each parent was used to inoculate YPD cultures that were grown overnight at 25 °C. The cultures were centrifuged at 5000×*g* for 5 min and the cells were washed and re-suspended in sterile H_2_O to a concentration of 10 g L^−1^. In a sterile 2 mL Eppendorf tube 100 µL of each suspension were added followed by 1 mL of YPD medium. The procedure was performed in triplicate. The tubes were vortexed and incubated statically for 5 days at 25 °C. Finally, the suspension was washed and re-suspended in 500 µL of starvation medium (0.1% yeast extract and 0.1% glucose) and incubated for 2 h at room temperature.

For selection of prototrophic hybrid cells, the suspension was vortexed and 100 µL were added per minimal selection agar (without lysine) plate. These plates were incubated at 37 °C (an inhibitory temperature for *S. eubayanus*) and the hybrids were selected based on the restoration of prototrophy and ability to grow at 37 °C.

### Confirmation of hybrid status

Colonies were checked for their hybrid status by rDNA-PCR amplification using the primers ITS1 (5′-TCCGTAGGTGAACCTGCGG-3′) and ITS4 (5′-TCCTCCGCTTATTGATATGC-3′), followed by a digestion using *Hae*III (New England Biolabs, Ipswich, MA, USA) [25]. Hybrids can be identified by having the ITS regions of both parents and thus the restriction profile appears as a combination of the parental profiles. Briefly, *S. cerevisiae* yields a 4 band pattern (320, 225, 180, 140 bp), *S. eubayanus* 3 bands (490, 225, 140 bp) and interspecific hybrids 5 bands (490, 320, 225, 180 and 140 bp). In addition, strain specific primers were used to confirm the presence of DNA from both parents, *MEX67* for *S. cerevisiae* 59A, ScerF2 (5′-GCGCTTTACATTCAGATCCCGAG-3′) and ScerR2 (5′-TAAGTTGGTTGTCAGCAAGATTG-3′), and a putative maltose transporter for *S. eubayanus* C902, SeubF4 (5′-CGATGAAGGGCTTATCCTCACTG-3′) and SeubF5 (5′-CGAGATGGTGTGCTTCGCC-3′).

### Ploidy determination by FACS

The DNA content was determined by flow cytometry essentially as described by Haase and Reed [21]. The parental strain *S. cerevisiae* 59A forms aggregates that impair an accurate measurement of the ploidy and so a strains deleted on *AMN1* gene was used [34]. Cells were grown overnight in YPD medium (1% yeast extract, 2% peptone, 2% glucose), and approximately 1 × 10^7^ cells were washed with 1 mL of 50 mM citrate buffer. Cells were fixed with cold 70% ethanol and incubated at room temperature for 1 h. Cells were then washed with 50 mM citrate buffer, resuspended in 50 mM citrate buffer containing 0.25 mg mL^−1^ RNAse A and incubated overnight at 37 °C. 1 mg mL^−1^ of Proteinase K was then added, and cells were incubated for 1 h at 50 °C. Cells were then stained with SYTOX Green (2 μM; Life Technologies, USA), and their DNA content was determined using a FACSAria cytometer (Becton–Dickinson). DNA contents were calculated by comparing fluorescence intensities with those of *S. cerevisiae* haploid (CEN.PK113-1A) and diploid (CEN.PK) reference strains. Measurements were performed on duplicate independent yeast cultures, and 100,000 events were collected per sample during flow cytometry.

### Origin of mitochondria

The origin of the mitochondria inherited by the hybrids was assessed by sequencing of the *COX2* gene. The gene was amplified by PCR using the following primers: COX2 (forward): GGTATTTTAGAATTACATGA and COX2 (reverse): ATTTATTGTTCRTTTAATCA as described in Ref. [7]. PCR products were purified with QIAquick PCR purification kit (Qiagen) and sequenced by GATC-Biotech using the same primers. Gene sequences were aligned using MUltiple Sequence Comparison by Log-Expectation (MUSCLE; http://www.ebi.ac.uk/Tools/msa/muscle/). The functionality of mitochondria was assessed by plating a fresh colony onto a YP plate containing 3% (w/v) glycerol as the sole carbon source.

### Temperature tolerance

The range of growing temperatures was determined by drop test. A culture of each strain was grown overnight in YPD medium. The OD_600nm_ of each culture was adjusted to 0.5 and three consecutive tenfold dilutions were made (0.05, 0.005 and 0.0005). 5 µL of each dilution were spotted in several plates and these were incubated at temperatures of 7, 10, 20, 28 and 37 °C and allowed to grow for 2 days at 28 and 37 °C, 3 days at 20 °C, 5 days at 12 °C and 7 days at 4 °C.

### Fructose and glucose uptake

For fructose and glucose uptake measurement, the yeast strains were grown at 20 °C in liquid YP medium containing glucose (2% w/v) to an OD_600nm_ between 4 and 8. The cells were harvested by centrifugation (10 min, 5000 rpm, 0 °C), washed with ice-cold water and 0.1 M tartrate-Tris (pH 4.2) and re-suspended in the same buffer to a concentration of 200 mg fresh yeast mL^−1^. Zero-trans rates of [U-^14^C]-fructose and [U-^14^C]-glucose uptake at 20 °C were determined with 5 mM of substrate with 10 s incubation time [33]. Two incubation times were tested to ensure linearity with respect to time with t2 corresponding to at least 90% of t1 value. [U-^14^C]-Fructose (ART0329) was from American Radiolabelled Chemicals (St. Louis, MO, USA) and [U-^14^C]-glucose (CFB 96) was from Amersham Biosciences (Buckinghamshire, UK).

### Apple juice fermentation

Apple juice fermentation was carried out with a similar setup as previously described for brewer’s wort [16, 47]. A concentrated mixture of apple and pear juice (74%; Finlandia cider, Senson Oy, Lahti, Finland) was used as substrate for cider fermentations. The concentrate contains sulphur dioxide, ammonium phosphate and the sweetener acesulphame K. Juice was diluted to a concentration of approximately 11 °Brix (64 g L^−1^ fructose and 32 g L^−1^ glucose). 1.5 L of 11 °Brix juice was fermented with selected strains in stainless steel cylindroconical vessels (so-called tall tubes) with dimensions of 6 cm internal diameter × 100 cm height (two litres) at a pitching rate of 3 g of fresh yeast L^−1^. Starting cultures were prepared by inoculating a fresh colony in 50 mL YPD and grown for 24 h at 25 °C, 150 rpm. The culture was then diluted in 500 mL 11 °Brix apple juice to an initial OD of 0.1 and grown for 2 days. The fermentations were carried out at 10 and 20 °C until no change in the residual extract was observed for 24 h.

Samples were withdrawn daily, centrifuged at 9000 rpm for 10 min at 1 °C and the pellet was washed with water, weighed, re-suspended in water and used for yeast analyses. Dry yeast masses were determined by drying the washed yeast slurry overnight at 105 °C in a pre-weighed porcelain crucible. Specific gravity, alcohol level and pH were determined from the centrifuged and degassed fermentation samples using an Anton Paar density meter DMA 5000 M with Alcolyzer Beer ME and pH ME modules (Anton Paar GmbH, Austria).

Concentration of fermentable sugars (glucose and fructose) was measured by HPLC using a waters 2695 separation module and waters system interface module liquid chromatograph coupled with a waters 2414 differential refractometer (Waters Co., Milford, MA, USA). An Aminex HPX-87H organic acid analysis column (300 × 7.8 mm, Bio-Rad) was equilibrated with 5 mM H_2_SO_4_ (Titrisol, Merck, Germany) in water at 55 °C and samples were eluted with 5 mM H_2_SO_4_ in water at a 0.3 ml min^−1^ flow rate.

### Quantification of volatile compounds

Fourteen yeast-derived volatile compounds were determined by headspace gas chromatography with flame ionisation detector (HSGC-FID) analysis. Four-millilitre samples were filtered (0.45 μm) and incubated at 60 °C for 30 min, and then 1 mL of gas phase was injected (split mode; 225 °C; split flow of 30 mL min^−1^) into a gas chromatograph equipped with an FID detector and headspace autosampler (Agilent 7890 Series; Palo Alto, CA, USA). Analytes were separated on a HP-5 capillary column (50 m × 320 μm × 1.05 μm column; Agilent, USA). The carrier gas was helium (constant flow of 1.4 mL min^−1^). The temperature programme was 50 °C for 3 min, 10 °C min^−1^–100 °C, 5 °C min^−1^–140 °C, 15 °C min^−1^–260 °C and then isothermal for 1 min. Compounds were identified by comparison with authentic standards and were quantified using standard curves. 1-Butanol was used as internal standard.

Twelve sulphur volatile compounds (SVC) in the ciders produced at 20 °C were quantified by Campden BRI (Nutfield, Surrey, UK) by purge-and-trap linked to GC with chemiluminescence detection. The sample was purged with helium into a Tekmar–Dohrmann sample concentrator then the trapped volatiles were desorbed and injected into the GC coupled with a sulphur-specific Sievers chemiluminescence detector. Sulphur volatiles were individually calibrated against reference standards.

### Consumer study

To assess the quality of the ciders produced by the hybrids relative to the parents, a panel of volunteer consumers rated the pleasantness of aroma on a scale of 1 (dislike extremely) to 9 (like extremely) of the ciders produced at 20 °C. The panel consisted of 45 women and 13 men between 18 and 65 years old from the southwest of Finland. Data were collected using Compusense Cloud (Compusense Inc. Ontario, Canada). The study was conducted at the sensory laboratory of the Functional Foods Forum (ISO 8589), University of Turku.

### Data analysis

Statistical analysis was performed with R (http://www.rproject.org/; v3.3.0) using one-way ANOVA and Tukey test. Visualisation of data in heat-maps was done with the package ggplot based on *z* scores. The *z* scores (*z*) were calculated as *z* = (*x*−*μ*)/*σ*, where *x* is the concentration of an aroma compound in a particular cider sample, *μ* is the mean concentration of that aroma compound in all ciders, and *σ* is the standard deviation of concentration of that aroma compound in all ciders.

## Results

### Confirmation of hybrid status, ploidy and mitochondria determination

The colonies growing in selective conditions were regarded as potential hybrids and were screened through ITS PCR followed by digestion with *Hae*III and species specific PCR. The 5 band digestion pattern (Fig. S1a) and the 2 band amplification (Fig. S1b) confirmed the presence of genetic material from the two different species. Additionally, the presence of chromosomes from both parents can be seen in the karyotype (Fig. S1c).

The ploidy measurements confirmed that *S. cerevisiae* 59A is haploid (1.01*n*) and *S. eubayanus* diploid (2.07*n*) (Fig. S2, Table 1). The hybrids were found to be roughly diploid but with small variation in DNA content between them, ranging from 2.07*n* to 2.38*n* (Fig. S2, Table 1). Sequencing of *COX2* gene revealed that hybrids C962, C963 and C964 contain *S. cerevisiae* mitochondrial DNA (Table 1; Fig S3). PCR using DNA from the strains C965, C966 and C967 did not yield any product in several attempts, suggesting defective or absent mitochondria. The functionality of the mitochondria was evaluated by testing the ability to grow in medium YP containing glycerol as carbon source. This assay confirmed the inability of hybrids C965–C967 to grow on non-fermentable carbon sources indicating the absence of functional mitochondria (Fig. S4).

### Temperature tolerance and uptake of the hexoses glucose and fructose

Growth temperature and sugar uptake are the major factors affecting the fermentation rate. We tested the range of growth temperatures by drop test and the uptake of radiolabelled glucose and fructose.

**Fig. 1:**
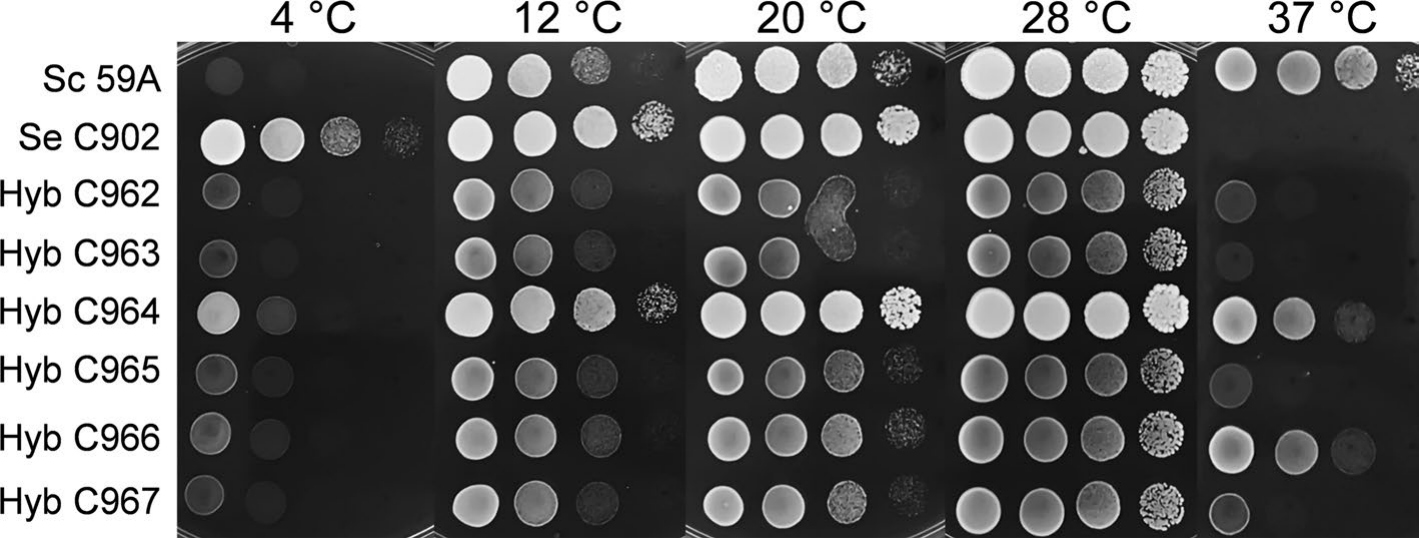
Drop test for assessment of the range of growth temperatures of the hybrids (C962–C967) relative to the parents (59A, C902). Plates were grown for 2 days at 28 and 37 °C, 3 days at 20 °C, 5 days at 12 °C and 7 days at 4 °C

The strains reacted differently to incubation temperatures (Fig. 1). *S. cerevisiae* 59A preferred the highest temperatures (>28 °C) while *S. eubayanus* grew well up to 28 °C but not further with particularly good ability to grow at 4 °C. Hybrid strains grew in the whole range of temperatures tested, however, with marked differences. Strains C964 and C966 displayed the highest versatility (Fig. 1).

**Fig. 2:**
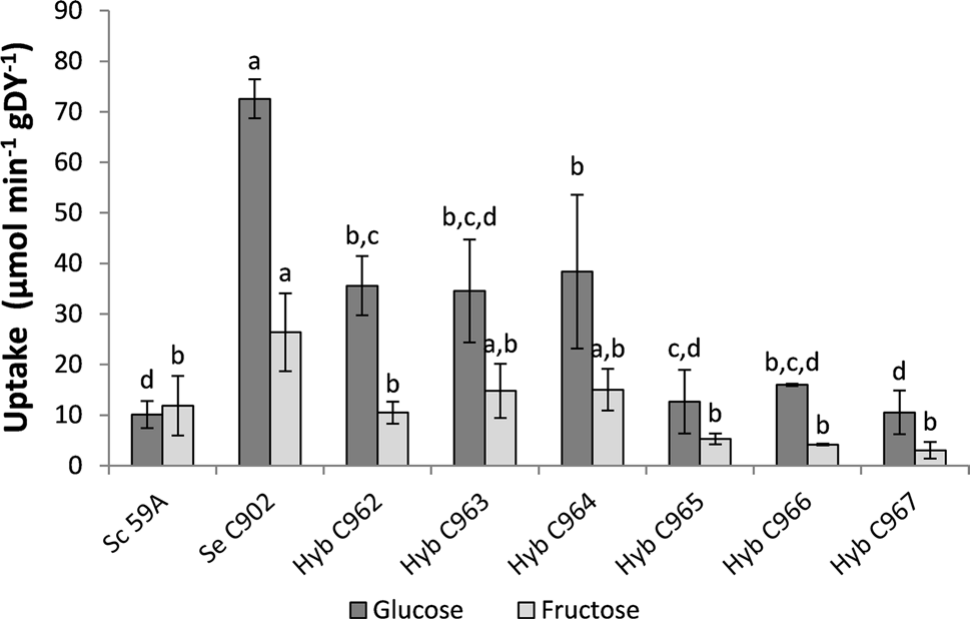
Zero-trans rates of glucose and fructose uptake activity (µmol min^−1^ g^−1^ DY) of the strains in study, measured at 20 °C. 59A and C902 are *S. cerevisiae* and *S. eubayanus* parents, respectively and C962–C967 are hybrid strains. Values are means of three independent assays; *error bars* represent standard deviation; different letters (*a*–*d*) represent significant differences between strains for a given sugar as determined by one-way ANOVA and Tukey test

The uptake of the sugars present in apple and pear juice, glucose and fructose, showed that *S. cerevisiae* parent had a similarly low ability to take up both of the sugars (Fig. 2). In contrast, *S. eubayanus* C902 had the highest uptake activity for both sugars, and the uptake of glucose was more than two times higher than that of fructose. The ability of the hybrids to take up sugars varied from 10.5 to 38.4 and 3.0 to 15.0 µmol min^−1^ g DY^−1^ for glucose and fructose, respectively, with ratios between glucose and fructose uptake higher than 2. At this stage, three hybrid strains were chosen for further characterization based on uptake activity, C967 with the lowest activity, C962 with intermediate activity and C964 with the highest activity.

### Fermentation in cider-making conditions

**Fig. 3:**
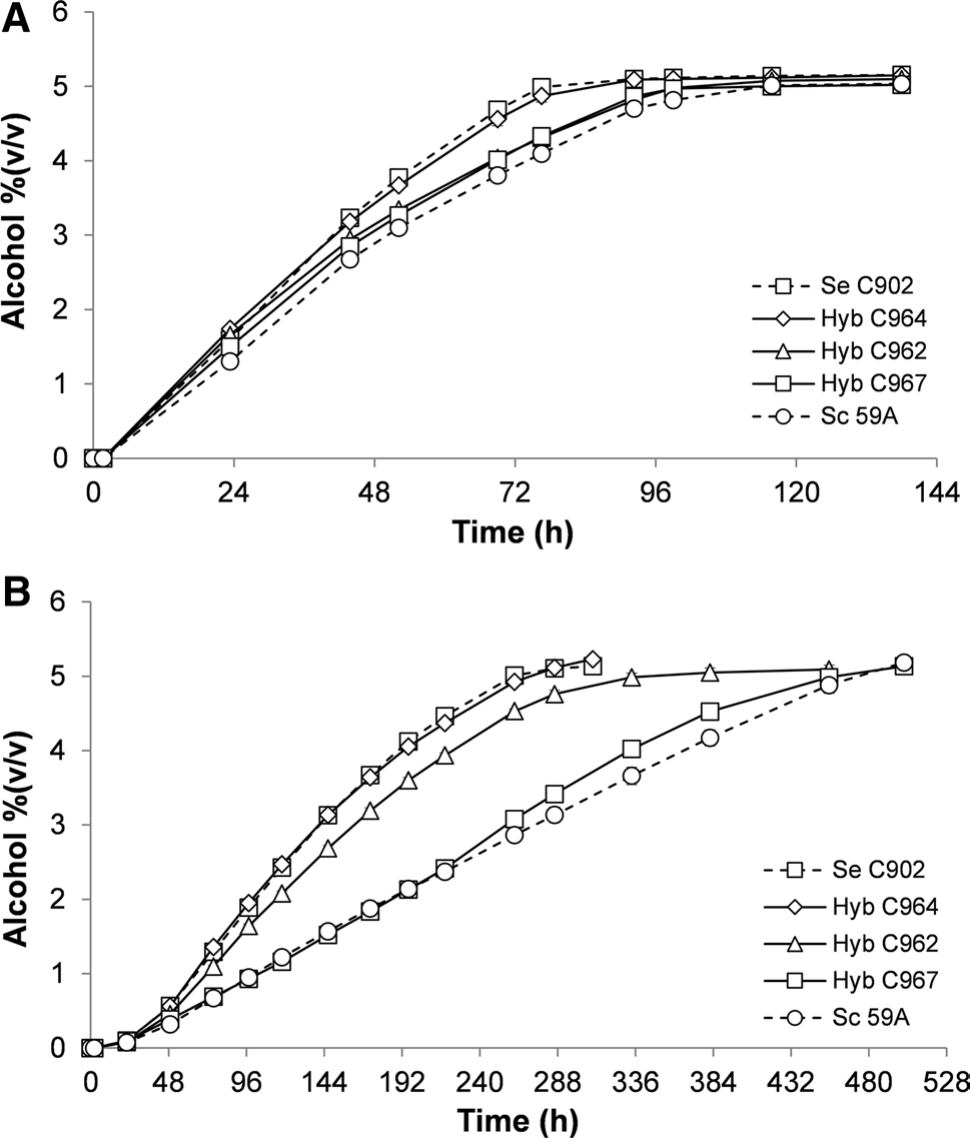
Fermentation (% ABV) of 11 °P apple juice at 20 °C (**a**) and 10 °C (**b**). Values are means from two independent fermentations and *error bars* where visible represent the range

All strains were able to completely ferment the juice to about 5% ABV (Alcohol by volume), although at different rates. In accordance with the higher uptake rates of both glucose and fructose, at 20 °C *S. eubayanus* and C964 are able to complete the fermentation after 72 h (Fig. 3a). The hybrids C962 and C967 follow with a slower rate, fermenting all the available sugars at 96 h, while the *S. cerevisiae* 59A had the worst performance (Fig. 3a). By reducing the fermentation temperature to 10 °C the cold-tolerant nature of *S. eubayanus* becomes more evident. The fermentation time was considerably longer at the lower temperature and the differences between the strains were clearer. The hybrid C964 and *S. eubayanus* C902 were the fastest fermenters, completing after 288 h (Fig. 3b). The hybrid C962 here performed quite differently from C967, consuming all sugars in 384 h (Fig. 3b). The hybrid C967 and *S. cerevisiae* 59A took more than 480 h to ferment the juice (Fig. 3b). The variable sugar uptake rates of the hybrid strains (Fig. 2) also correlate well with the consumption of glucose and fructose (Fig. S3). At both temperatures tested, hybridization with *S. eubayanus* C902 resulted in an improvement of the fermentation performance in relation to the parent *S. cerevisiae* 59A.

### Cider quality assessment

**Fig. 4:**
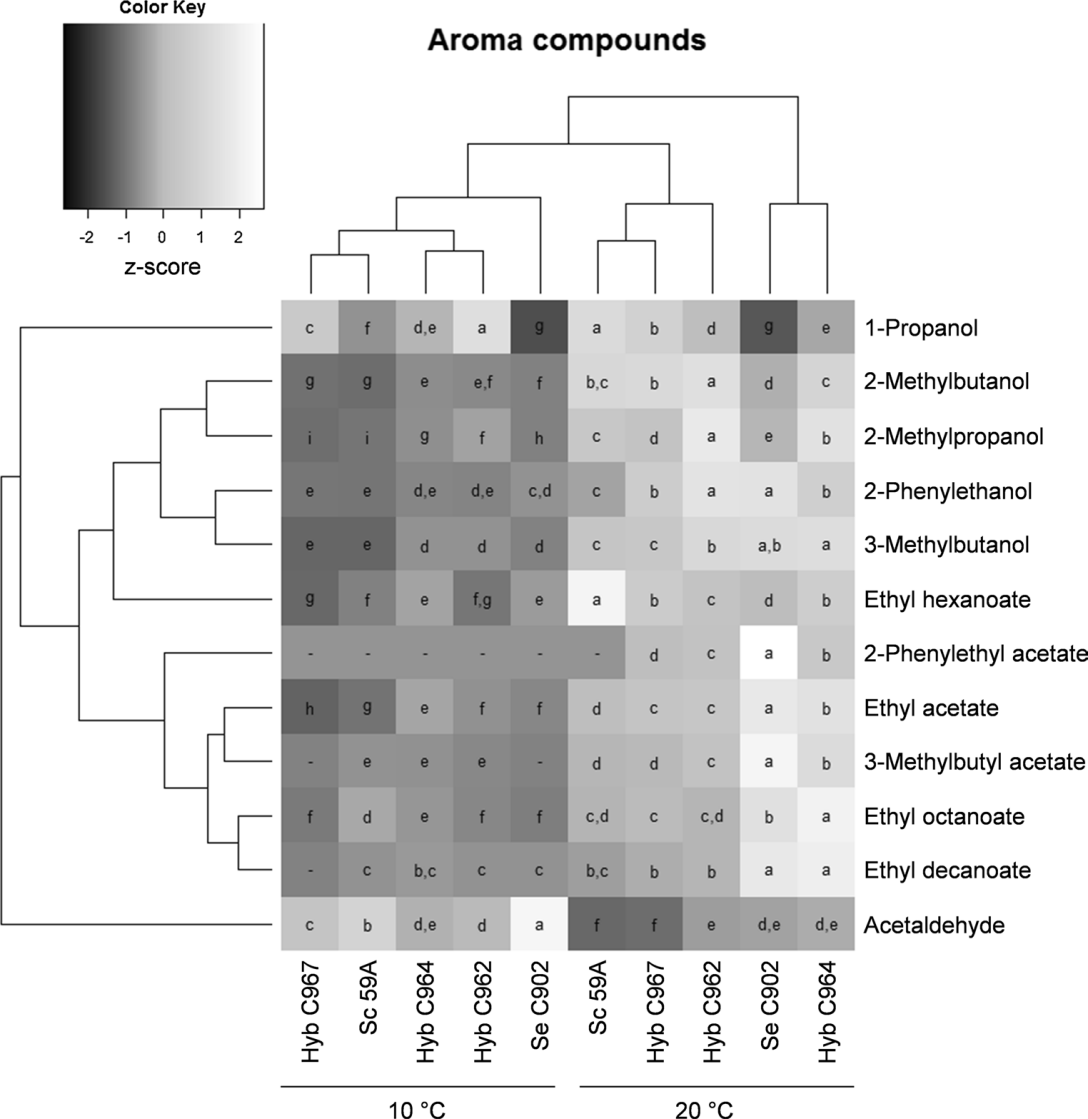
Heat-map representative of relative concentrations of aroma compounds in the ciders. The heat-map was generated based on *Z* scores; strains with different letters (*a* to *i*) for the same compound are significantly different; significance letters are ordered alphabetically from the highest to lowest concentration of each aroma compound

In the cider industry, improvement of fermentation performance is only advantageous if it does not come at the expense of product quality. With that in mind, we measured the concentration of esters and higher alcohols in the final ciders as well as unpleasant sulphuric volatile compounds. This was followed by an evaluation of the pleasantness of aroma of the produced ciders by a consumer panel. Aromatic profiles of the ciders produced at both temperatures were clearly different. A fermenting temperature of 20 °C resulted in an average of three times greater production of volatile compounds than at 10 °C, and 3 times lower production of acetaldehyde (Fig. 4; Table S1). Considering variations between strains, the hybrid C967 plotted closely to the *S. cerevisiae* parent, producing a similar aroma profile and generally producing the lowest amounts of esters and higher alcohols (Fig. 4; Table S1). Similarly, the aroma production by strain C962 at 10 °C is closer to that of C964 while at 20 °C it is closer to C967 and 59A. *S. eubayanus* C902 in general produced high levels of aroma compounds, particularly the rose-like 2-phenylethanol. The hybrid strain C964 once again performed similarly to *S. eubayanus*, producing very aromatic cider relative to the 59A *S. cerevisiae* strain (Fig. 4; Table S1). *S. eubayanus* C902 has shown to be a good fermenting strain and also has the ability to produce reasonably high amounts of desirable aromatic compounds but has the disadvantage of producing significant quantities of Sulphur Volatile Compounds (SVCs) that have low aroma perception thresholds and can mask the cider’s fruity/floral notes even when present in very low concentrations (Table 2). Of the twelve sulphur compounds measured, four were at concentrations below the detection limit (diethyl sulphide, hydrogen sulphide, n-propane thiol and t-butyl mercaptan) and four compounds were detected at concentrations below the odour threshold (dimethyl disulphide, ethyl thioacetate, ethylene sulphide and methyl thioacetate) and thus are unlikely to have a direct impact on the final product. The cider produced with *S. eubayanus* C902 contained diethyl disulphide and methanethiol at concentrations close to the odour threshold of 0.4 and 1.5 µg L^−1^, respectively. Ethanethiol was found to be present at more than two times the odour threshold in the ciders produced by *S. eubayanus*. These compounds are characterised by a strong unpleasant rotten cabbage and onion aroma. Interestingly, these seem to be absent or much reduced in the hybrid strains, as in the *S. cerevisiae* parent.

**Table 2:** Concentration of sulphur volatile compounds (SVC’s) in µg L^−1^ in the ciders produced at 20 °C (± standard deviation of two biological replicates)

	Odour threshold	Sc 59A	Se C902	Hyb C962	Hyb C964	Hyb C967
Diethyl disulphide	0.4^a^ [8]	n.d.	0.44 ± 0.16	n.d.	n.d.	n.d.
Dimethyl disulphide	20–45^c^ [38]	0.35 ± 0.07	0.35 ± 0.35	0.55 ± 0.07	0.65 ± 0.35	0.35 ± 0.07
Dimethyl trisulphide	0.2^c^ [20]	n.d.	n.d.	0.10 ± 0.14	0.15 ± 0.21	n.d.
Ethanethiol	1.1^c^ [38]	n.d.	2.34 ± 0.74	0.12 ± 0.16	n.d.	n.d.
Ethyl thioacetate	10^a^ [8]	n.d.	1.15 ± 0.35	n.d.	n.d.	n.d.
Ethylene sulphide		0.15 ± 0.07	0.05 ± 0.07	0.30 ± 0.00	0.20 ± 0.00	0.30 ± 0.14
Methanethiol	1.5^b^	0.20 ± 0.00	1.15 ± 0.50	1.10 ± 0.07	0.65 ± 0.07	0.30 ± 0.14
Methyl thioacetate	50^a^ [8]	n.d.	1.55 ± 0.50	1.50 ± 0.14	0.90 ± 0.14	0.30 ± 0.42

*n.d.* not detected, concentration below detection limit

^a^in beer

^b^in cider

^c^in wine

**Fig. 5:**
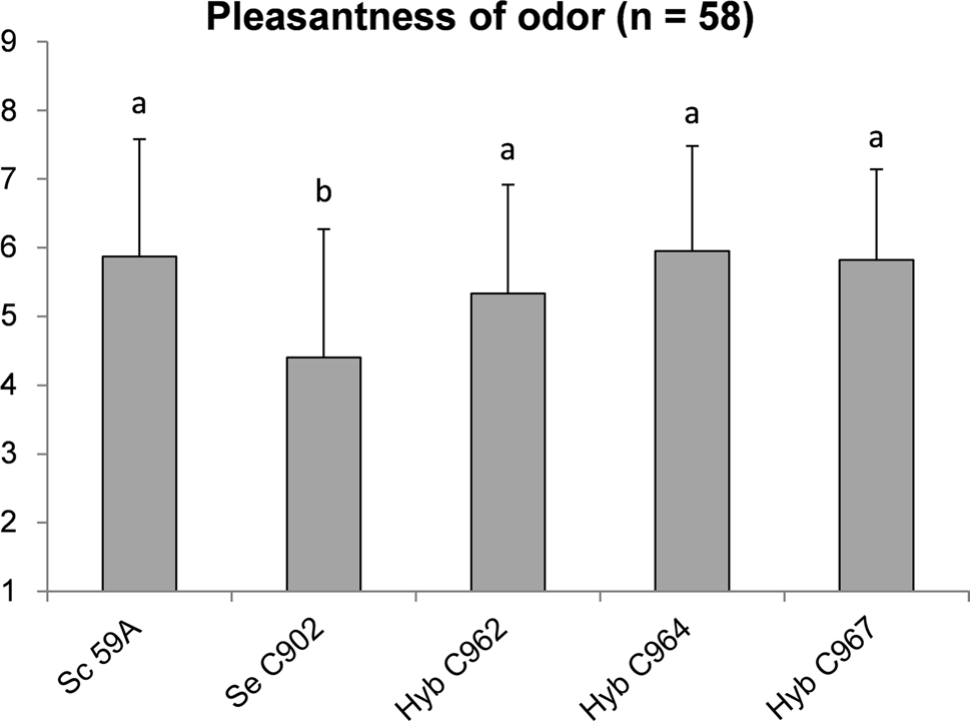
Pleasantness of the cider aroma as evaluated by a consumer panel. Ciders were rated from extremely unpleasant (*1*) to extremely pleasant (*9*).* Error bars* represent standard deviation; different letters (*a*, *b*) represent significant differences

To evaluate how the combination of the different measured compounds affects perceived quality of the ciders, a panel of 58 consumers was asked to rate the ciders based on the pleasantness of aroma. The results clearly show that the ciders produced by all the hybrids are as pleasant as the cider made with *S. cerevisiae* 59A, while being significantly more pleasant that the cider produced by *S. eubayanus* (Fig. 5). This confirms that not only was it possible to significantly reduce the production of SVCs through interspecific hybridization as the analytical data shows, but that this was also reduced to below sensory detection levels in the final product.

## Discussion

The use of hybrid yeast involving *S. eubayanus* is still limited to lager beer brewing. The lager yeast are, however, a group with rather low genetic and phenotypic diversity [26]. The limited aroma spectrum of lager strains is desirable for lager beers, where a clean aroma is generally preferred [26]. Ciders, however, benefit from a more complex profile. Wine strains represent a more diverse group of strains and therefore a more interesting alternative for cider making. After crossing a wine strain with *S. eubayanus*, hybrid strains were expected to inherit the more pleasant aroma profile of the former and the reportedly high tolerance to low temperatures of the latter [31].

We obtained diploid hybrid strains with variable performance in apple juice, with the best strain performing similarly to *S. eubayanus* and producing comparable amounts of aroma compounds but with the advantage of producing SVCs at concentrations under the detection level.

Hybrids generated by rare mating often contain the complete DNA of both parents [19, 24]; this was, however, not the case here as the hybrids were diploid rather than triploid. This may be either caused by the high sporulation capacity of *S. eubayanus* [48] that resulted in a spore of C902 mating with *S. cerevisiae* 59A, or by loss of chromosomes post-hybridization due to chromosome instability and even incompatibility. Examples of the latter are the chromosome III from *S. cerevisiae* that has high loss frequency [28] and the chromosome XII from *S. bayanus* being partially incompatible with the *S. cerevisiae* genome [30]. This phenomenon seems to be strain and species specific [28, 32]. *S. cerevisiae* × *S. uvarum* and *S. cerevisiae* × *S. kudriavzevii* hybrids show different rates of chromosome loss with hybrids involving *S. kudriavzevii* being more unstable [32]. The chromosomes from the non-*S. cerevisiae* parent tend to be have higher loss frequency [43, 44]. However, the ploidy of the *S. cerevisiae* × *S. eubayanus* hybrids generated in this study was measured shortly after purification and confirmation in the hybrid state and, thus, such rearrangements of the hybrid genome are unlikely to have had the chance to occur. The alternative, i.e., fusion of a haploid cell and haploid spore may therefore be more plausible. The diploid state may explain why only single parent transgression in fermentation was observed. Recent studies on artificial hybrids with different ploidies have shown a direct and positive relationship between DNA content and fermentation rate [24].

The phenotypic variations among hybrid strain were visible already in the range of growth temperatures. An ability to grow at high temperatures (37 °C), a property not possessed by *S. eubayanus* C902, was expected as this was one of the selection factors. However, at the lower range of 4–12 °C, the strain C964 was clearly different to the others. The mechanisms of cold tolerance of *S. eubayanus* are still unclear [26]. One possible explanation is that the genome of *S. eubayanus* may be more dominant in this hybrid strain which also shows a similar fermentation profile; however, that is not yet known. A higher proportion of *S. eubayanus* genetic material in lager yeast hybrids appears to relate with the cold tolerance of these strains [16].

The non-Mendelian nature of mitochondria inheritance can also influence the phenotypic properties of new hybrid strains. The hybrids described in this study either contain *S. cerevisiae* mitochondria (C962–C964) or no mitochondria was found (C965–C967). The hybrids containing *S. cerevisiae* mitochondria showed superior hexose transport, but the advantage to fermentation performance was only observed at low temperature fermentation (10 °C), possibly due to the reduced influence of mitochondria on fermentative behaviour [1]. However, *S. cerevisiae* × *S. uvarum* hybrids that retain the *S. cerevisiae* mitochondrial genome have lower expression of genes related to hexose transport and glycolysis/fermentation pathways [51]. Nevertheless, the same study also suggests that the inheritance of mitochondrial DNA is strain specific and therefore hybrids obtained from repeated crosses with the same parental strains will retain the same mtDNA [51]. This seems to be true for lager strains, where only *S. eubayanus* type mitochondria were found independently of the strain group [13, 45]. When hybrids are generated artificially, the origin of mitochondrial DNA varies even when using the same parental strains [24]. A more recent study showed that, in addition to the strain background, environmental conditions also influence the origin of the mtDNA retained in interspecific hybrids [23]. This is also, however, unlikely to explain the differences observed between the hybrids generated in this work, since these were maintained in similar conditions post-hybridization and during hybrid isolation. The most likely explanation for the loss of mitochondria may be that these 3 hybrids (C965–C967) initially contained *S. eubayanus* mitochondria, but during the hybrid isolation process the mitochondria were lost due to the high temperatures employed (37 °C). At this stage, the hybrid genomes are unstable and such temperature conditions may have induced the loss of *S. eubayanus* mitochondria, considering 37 °C is a restrictive temperature for *S. eubayanus*. Non-permissive temperatures have been shown to restrict the transmission of mitochondria in *S. cerevisiae* strains with mutations in *MDM1* and *MDM2* genes [36]. These results suggest that there is still work to do to understand which factors influence the inheritance and the retention of functional mitochondria in interspecies hybrids.

The sugar uptake kinetics of *S. eubayanus* C902 are superior to those of *S. cerevisiae* 59A and, while the hybrids show lower uptake rates than *S. eubayanus*, these are still generally more efficient than *S. cerevisiae* in transporting glucose and fructose and this is reflected in the fermentation performance. The mechanism responsible is, however, not clear as little is known about hexose transport kinetics of *S. eubayanus*. In *S. cerevisiae*, *HXT* transporters are induced/repressed at varying concentrations of glucose, i.e., low affinity *HXT1* and *HXT3* are induced by high glucose levels while high-affinity *HXT2* and *HXT4*-*7* are induced by low glucose concentration and are essential at the end of fermentation together with *FSY1* for fructose uptake [5, 14, 27, 29]. The latter gene is present in both parent strains and they share 78% similarity at the amino acid level [14]. The activity of Fsy1 symporter may be relevant for fructose scavenging in the later stages of fermentation as it is repressed at high fructose concentrations and induced by non-fermentable carbon sources such as ethanol [5, 14].

A disadvantage of using *S. eubayanus* for industrial fermentations is that it produces perceptible amounts of unpleasant sulphur volatiles. These completely masked the fruit and floral notes of the esters and higher alcohols, despite these being produced in much higher concentrations than in the *S. cerevisiae* fermentations. Unpleasant sulphur-like notes in beers produced with *S. eubayanus* strains have similarly been described before by Mertens and coworkers [37]. These were also apparently absent in hybrids produced with *S. cerevisiae* strains. Hybridization approaches for the removal of 4-vinylguaiacol (phenolic off-flavour) and H_2_S have likewise been described [11, 53]. The production of SVCs by *S. eubayanus* may be due to differential amino acid uptake. We noted that *S. eubayanus* C902 has greater nitrogen requirements than *S. cerevisiae* 59A and the hybrids (data not shown). In nitrogen-deficient conditions, the sulphate reduction sequence pathway is triggered to meet the yeast demand in cysteine and methionine, this results in accumulation of sulphide that is then released from the cells as H_2_S [52]. H_2_S reacts with ethanol or acetaldehyde to form ethanethiol which can be oxidised to diethyl disulphide [4, 46, 49] while methanethiol is formed by degradation of methionine [46].

Phenotypically, the hybrids display an interesting combination of the parental properties indicating potential for cider fermentation and possibly further applications. A wider range of temperatures permitting growth could be used to modulate the properties of the final product. It was shown that the aromatic profile is substantially different at fermentation temperatures of 10 and 20 °C in accordance with what is observed in brewing fermentations with natural lager hybrids [16]. Mass production of these yeast strains may also be facilitated as tolerance of temperatures as high as 37 °C is likely to facilitate production in the form of active dried yeast [50]. In addition, the high hexose uptake rates suggest that *S. eubayanus* and the hybrids could be advantageous for low-temperature fermentation of other sugar-rich substrates such as grape must.

## Electronic supplementary material

Below is the link to the electronic supplementary material.

Supplementary material 1 (DOCX 12 kb)

Supplementary material 2 (DOCX 15 kb)
