# Supplementary material for: Improved cider fermentation performance and quality with newly generated Saccharomyces cerevisiae × Saccharomyces eubayanus hybrids
Source: J Ind Microbiol Biotechnol. 2017 Apr 27;44(8):1203–13. doi: 10.1007/s10295-017-1947-7 (PMC5511608; doi:10.1007/s10295-017-1947-7)
Supplement: Supplementary file 1 — Supplementary material 1 (DOCX 12 kb) [file 10295_2017_1947_MOESM1_ESM.docx]

**SUPPLEMENTARY FIGURES LEGENDS**

Figure S1. Confirmation of hybrid status by ITS amplification, and digestion with *HaeIII* (A), species specific PCR (B) and karyotyping (C).

Figure S2. (A) DNA content of a *S. cerevisiae* haploid (CEN.PK113-1A; blue) and diploid (CEN.PK; green) reference strains. (B) *S. cerevisiae* 59A (red) and *S. eubayanus* C902 (purple). (B) *S. cerevisiae* 59A × *S. eubayanus* C902 hybrids.

Figure S3. Sequence alignment of *COX2* genes from *S. cerevisiae* 59A, *S. eubayanus* C902 and the hybrids (C962-C964).

Figure S4. Evaluation of the ability to grow on YP medium with 3 % glycerol of the strains in study.

Figure S5. Time profile of fructose and glucose consumption (g.L^-1^) and alcohol production (% v/v) throughout the fermentation. Values represent the mean of two independent fermentations and error bars the standard deviation. Circles represents fructose, triangles glucose and squares with dashed lines alcohol concentrations.
