# Supplementary material for: Improved cider fermentation performance and quality with newly generated Saccharomyces cerevisiae × Saccharomyces eubayanus hybrids
Source: J Ind Microbiol Biotechnol. 2017 Apr 27;44(8):1203–13. doi: 10.1007/s10295-017-1947-7 (PMC5511608; doi:10.1007/s10295-017-1947-7)
Supplement: Supplementary file 2 — Supplementary material 2 (DOCX 15 kb) [file 10295_2017_1947_MOESM2_ESM.docx]

**SUPPLEMENTARY TABLE**

Table S1. Concentration of aroma compounds in the ciders (± standard deviation). Strains with different letters (a to i) for the same compound are significantly different; significance letters are ordered alphabetically from the highest to lowest concentration of each aroma compound.

|  | Sc 59A | | Se C902 | | Hyb C962 | | Hyb C964 | | Hyb C967 | |
| --- | --- | --- | --- | --- | --- | --- | --- | --- | --- | --- |
|  | 20 °C | 10 °C | 20 °C | 10 °C | 20 °C | 10 °C | 20 °C | 10 °C | 20 °C | 10 °C |
| Acetaldehyde | 1.73 (±0.10)^f^ | 27.14 (±2.77)^b^ | 11.54 (±0.22)^d,e^ | 43.60 (±4.15)^a^ | 10.79 (±0.22)^e^ | 15.62 (±0.96)^d^ | 13.45 (±0.69)^d,e^ | 14.45 (±0.74)^d,e^ | 2.69 (±0.06)^f^ | 19.86 (±2.01)^c^ |
| 2-Phenylethanol | 38.29 (±5.99)^c^ | 13.13 (±6.68)^e^ | 109.60 (±4.74)^a^ | 28.61 (±3.49)^c,d^ | 111.12 (±6.75)^a^ | 21.31 (±5.49)^d,e^ | 79.78 (±5.53)^b^ | 22.12 (±7.03)^d,e^ | 75.51 (±5.90)^b^ | 13.69 (±3.90)^e^ |
| 1-Propanol | 18.04 (±0.27)^a^ | 10.86 (±0.21)^f^ | 7.50 (±0.12)^g^ | 6.99 (±0.19)^g^ | 13.48 (±0.17)^d^ | 18.73 (±0.66)^a^ | 12.11 (±0.26)^e^ | 12.79 (±0.26)^d,e^ | 16.64 (±0.20)^b^ | 15.28 (±0.94)^c^ |
| 2-Methylpropanol | 47.83 (±0.65)^c^ | 7.54 (±0.32)^i^ | 33.10 (±0.97)^e^ | 12.14 (±0.12)^h^ | 86.63 (±2.33)^a^ | 24.91 (±0.34)^f^ | 77.62 (±0.45)^b^ | 17.27 (±0.46)^g^ | 43.46 (±3.81)^d^ | 4.54 (±0.35)^i^ |
| 2-Methylbutanol | 32.78 (±1.87)^b,c^ | 3.71 (±0.17)^g^ | 16.29 (±0.48)^d^ | 7.42 (±0.14)^f^ | 37.10 (±0.60)^a^ | 9.18 (±0.11)^e,f^ | 31.12 (±0.52)^c^ | 9.67 (±0.29)^e^ | 33.97 (±1.13)^b^ | 4.55 (±0.29)^g^ |
| 3-Methylbutanol | 107.29 (±1.93)^c^ | 13.41 (±0.71)^e^ | 161.81 (±5.06)^a,b^ | 38.53 (±2.21)^d^ | 153.64 (±4.24)^b^ | 51.93 (±0.56)^d^ | 166.69 (±4.13)^a^ | 51.40 (±1.23)^d^ | 112.38 (±13.92)^c^ | 13.13 (±1.03)^e^ |
| *Total higher alcohols* | *205.93 (±2.78)* | *35.52 (±0.82)* | *218.69 (±5.18)* | *65.08 (±2.22)* | *290.85 (±4.88)* | *104.74 (±0.94)* | *287.54 (±4.19)* | *91.13 (±1.37)* | *206.46 (±14.48)* | *37.50 (±1.47)* |
| 2-Phenylethyl acetate | n.d. | n.d. | 0.52 (±0.02)^a^ | n.d. | 0.13 (±0.01)^c^ | n.d. | 0.17 (±0.00)^b^ | n.d. | 0.10 (±0.01)^d^ | n.d. |
| 3-Methylbutyl acetate | 0.05 (±0.00)^d^ | 0.02 (±0.00)^e^ | 0.21 (±0.01)^a^ | n.d. | 0.07 (±0.00)^c^ | 0.01 (±0.01)^e^ | 0.14 (±0.01)^b^ | 0.02 (±0.00)^e^ | 0.05 (±0.01)^d^ | n.d. |
| Ethyl acetate | 3.79 (±0.12)^d^ | 1.20 (±0.08)^g^ | 8.00 (±0.40)^a^ | 1.86 (±0.11)^f^ | 4.59 (±0.16)^c^ | 2.20 (±0.07)^f^ | 7.24 (±0.18)^b^ | 2.90 (±0.07)^e^ | 4.41 (±0.40)^c^ | 0.64 (±0.06)^h^ |
| Ethyl hexanoate | 0.13 (±0.01)^a^ | 0.02 (±0.00)^f^ | 0.05 (±0.00)^d^ | 0.04 (±0.00)^e^ | 0.06 (±0.00)^c^ | 0.02 (±0.00)^f,g^ | 0.08 (±0.00)^b^ | 0.04 (±0.00)^e^ | 0.07 (±0.01)^b^ | 0.01 (±0.00)^g^ |
| Ethyl octanoate | 0.03 (±0.00)^c,d^ | 0.03 (±0.00)^d^ | 0.09 (±0.01)^b^ | 0.01 (±0.00)^f^ | 0.03 (±0.00)^c,d^ | 0.01 (±0.00)^f^ | 0.11 (±0.01)^a^ | 0.02 (±0.00)^e^ | 0.04 (±0.00)^c^ | 0.00 (±0.00)^f^ |
| Ethyl decanoate | 0.01 (±0.00)^b,c^ | 0.00 (±0.00)^c^ | 0.03 (±0.00)^a^ | 0.00 (±0.00)^c^ | 0.01 (±0.00)^b^ | 0.00 (±0.00)^c^ | 0.04 (±0.00)^a^ | 0.00 (±0.00)^b,c^ | 0.01 (±0.00)^b^ | n.d. |
| *Total esters* | *4.01 (±0.12)* | *1.26 (±0.08)* | *8.91 (±0.40)* | *1.90 (±0.11)* | *4.89 (±0.16)* | *2.24 (±0.07)* | *7.78 (±0.18)* | *2.98 (±0.07)* | *4.69 (±0.40)* | *0.65 (±0.06)* |
